# Supplementary figures and images for: A MAP1B–cortactin–Tks5 axis regulates TNBC invasion and tumorigenesis
Source: J Cell Biol. 2024 Feb 14;223(3):e202303102. doi: 10.1083/jcb.202303102 (PMC10866687; doi:10.1083/jcb.202303102)

Source data of Figure 1

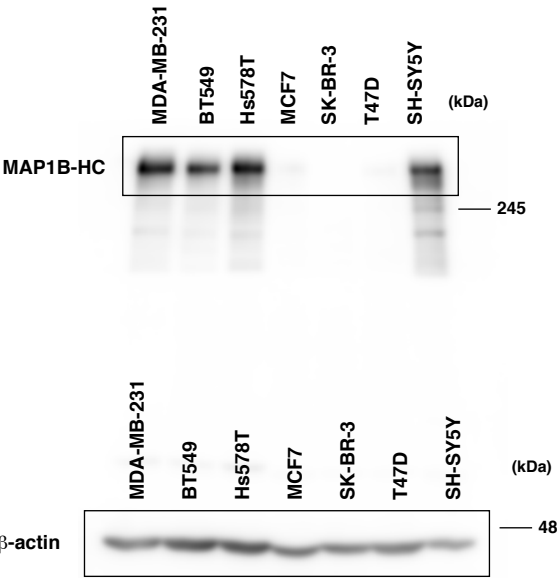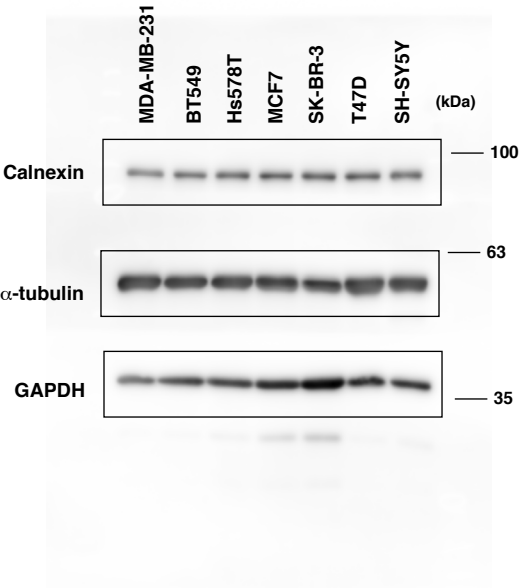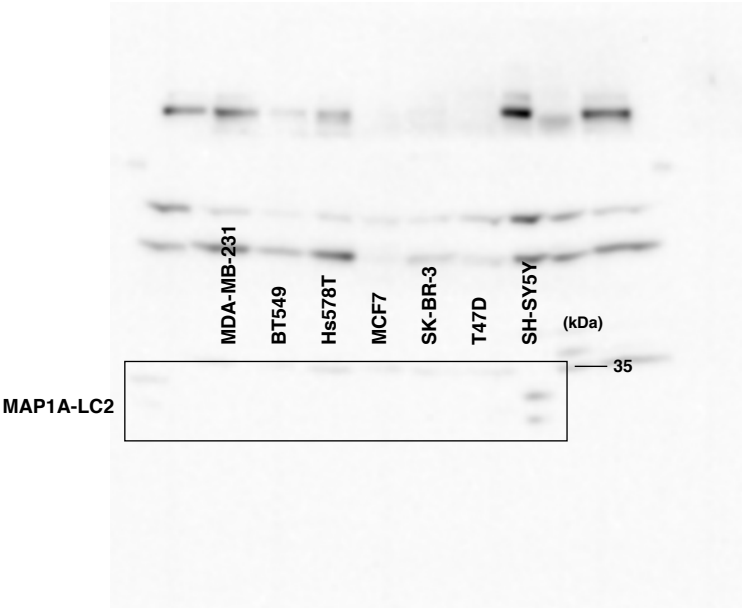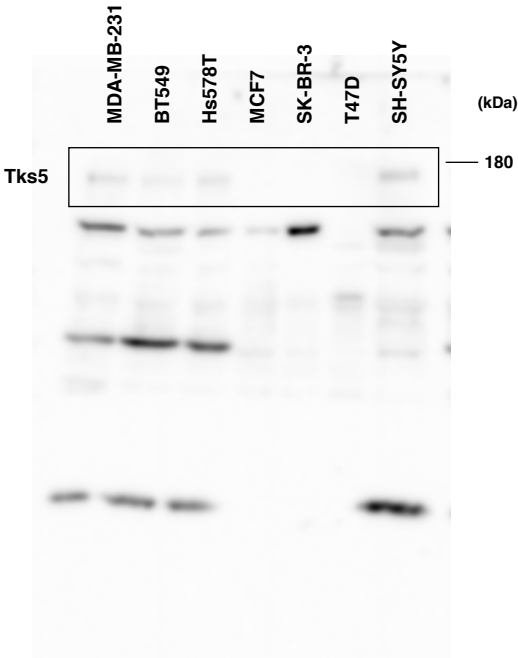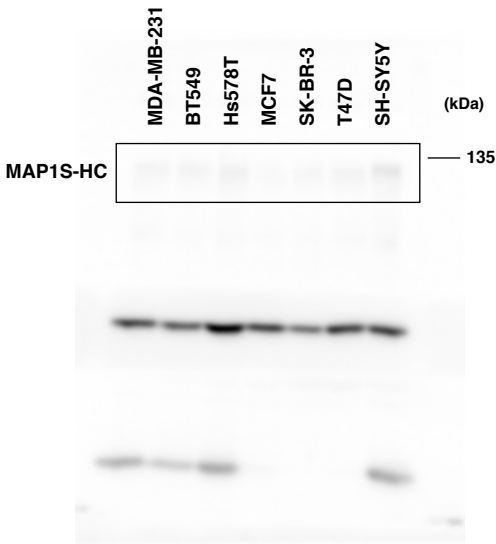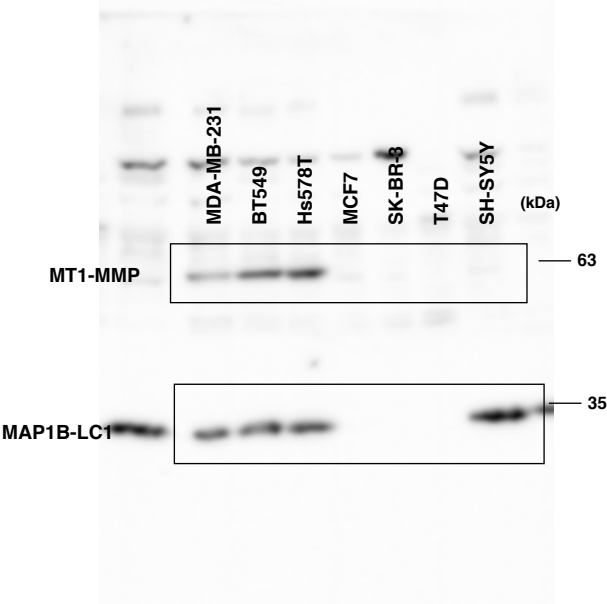

Supplement: SourceData F1 — is the source file for Fig. 1. [file JCB_202303102_SourceDataF1.pdf]

Source data of Figure 2

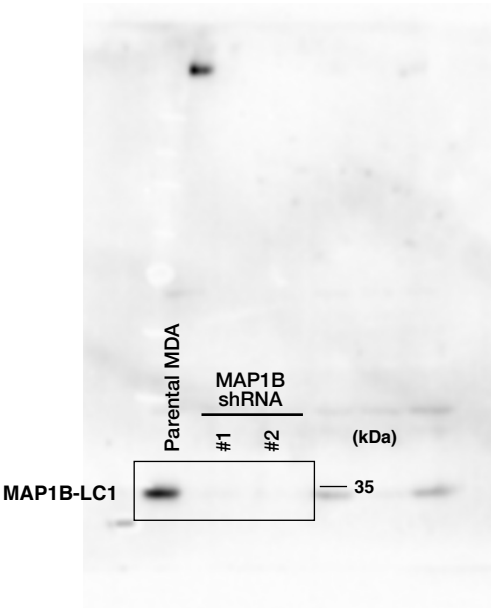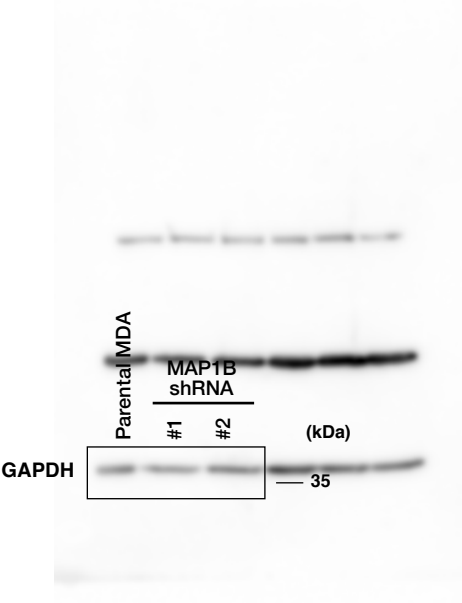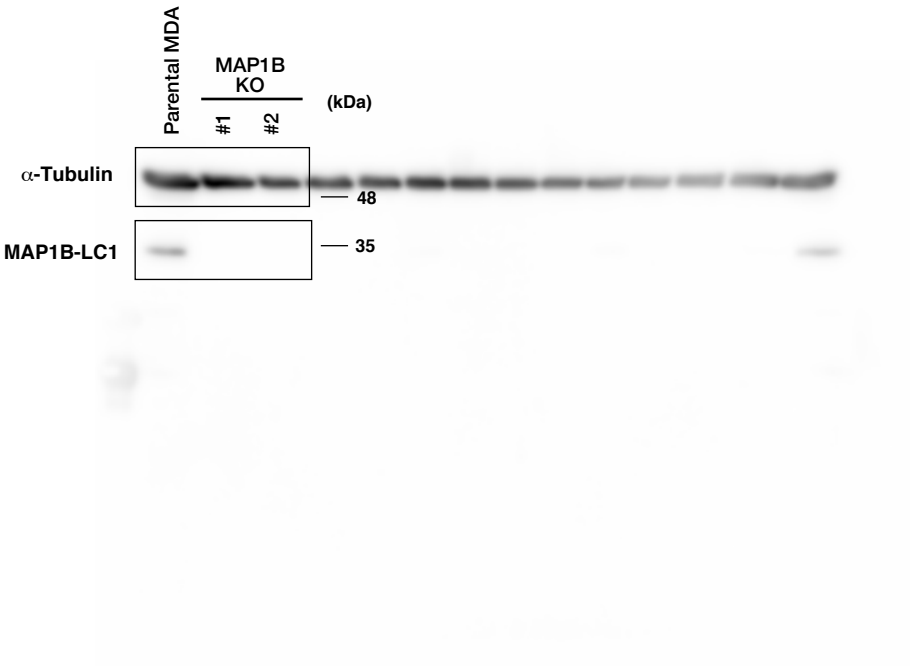

Supplement: SourceData F2 — is the source file for Fig. 2. [file JCB_202303102_SourceDataF2.pdf]

Source data of Figure 3

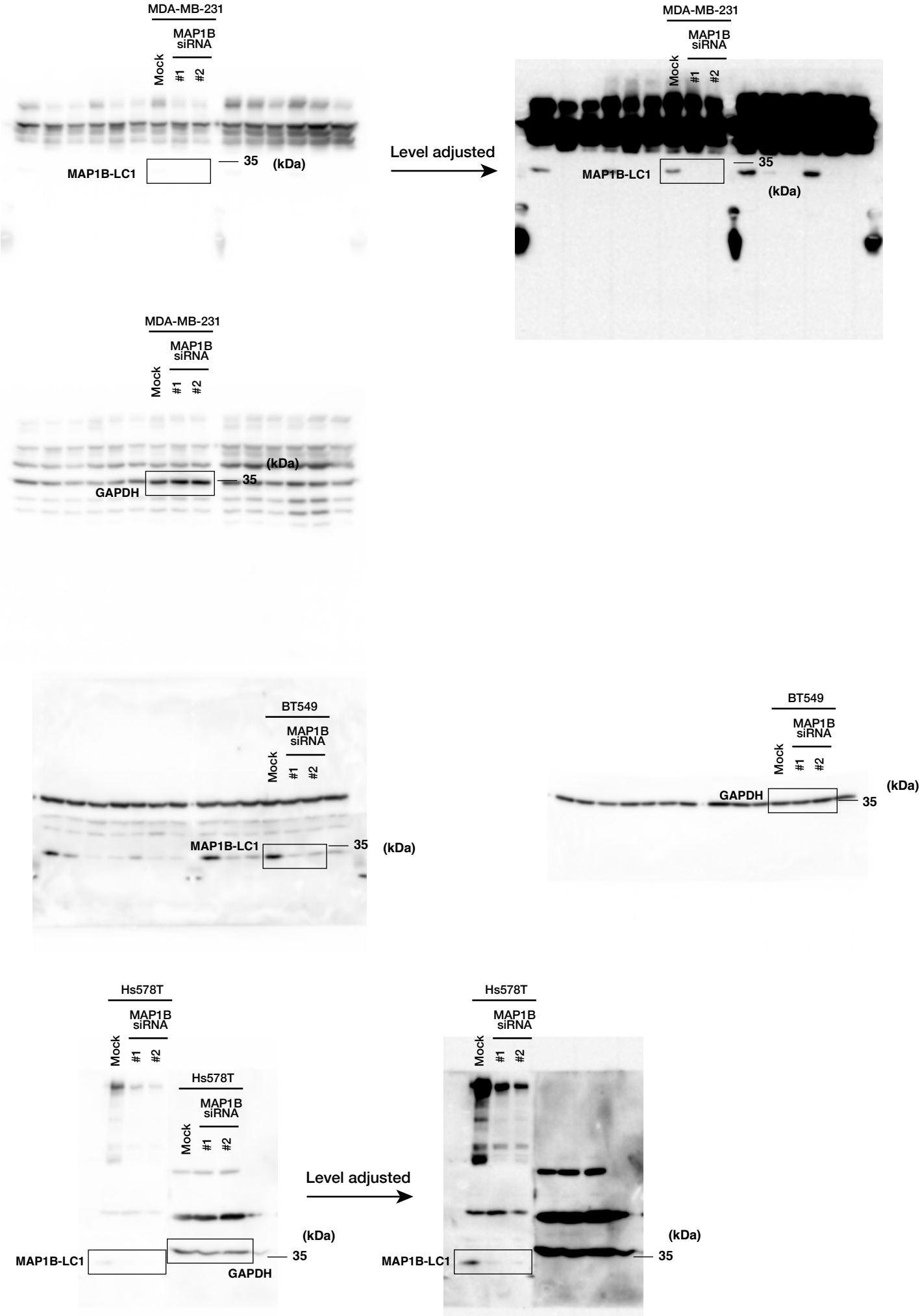

Supplement: SourceData F3 — is the source file for Fig. 3. [file JCB_202303102_SourceDataF3.pdf]

## Source data of Figure 4

**B**

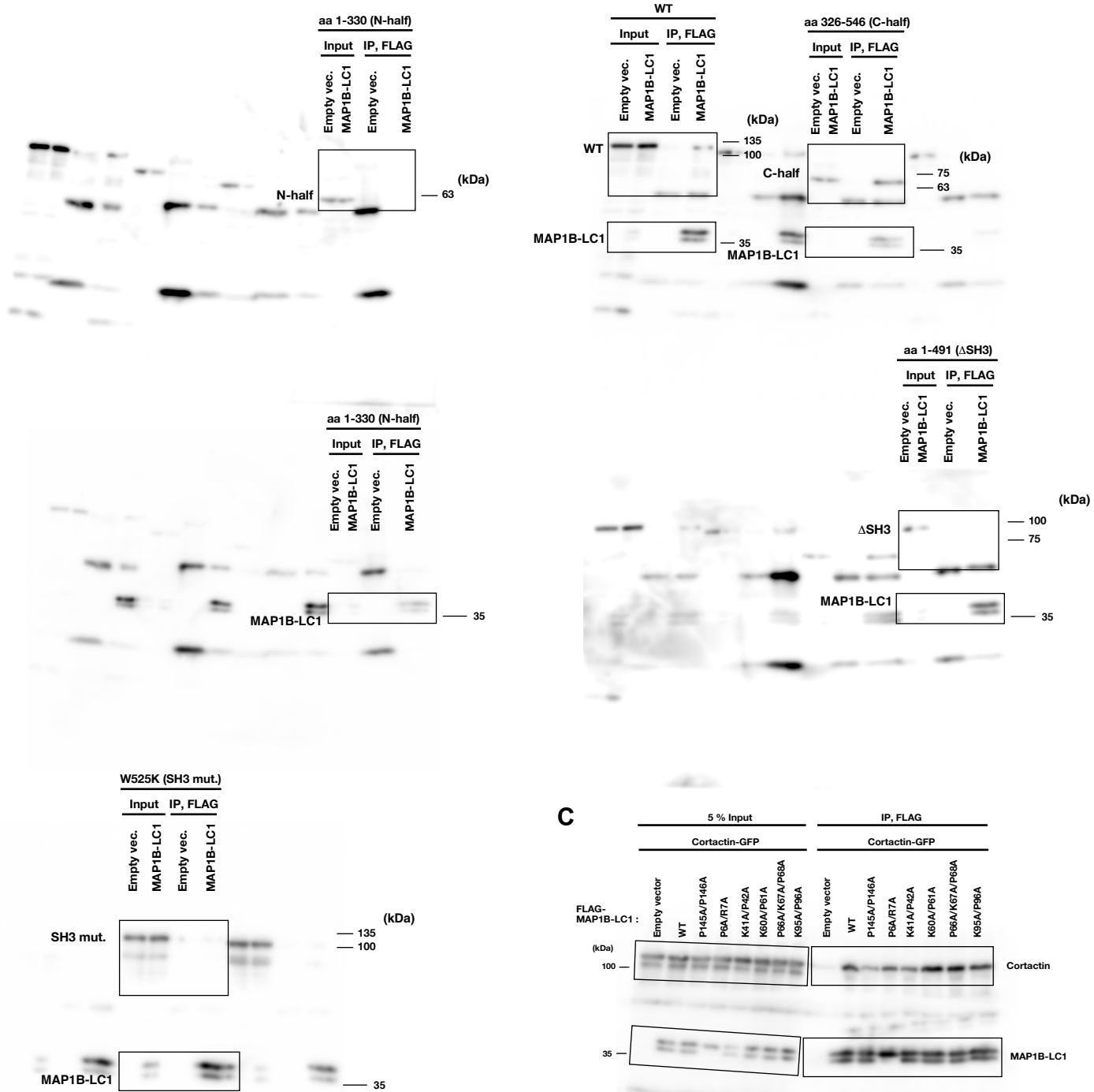

H

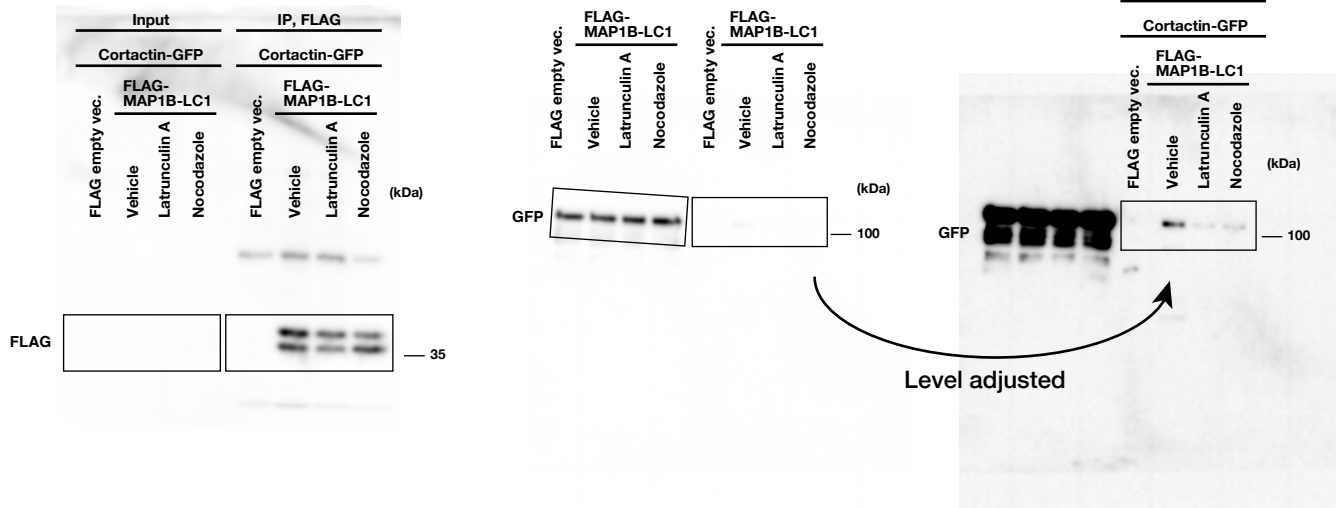

Supplement: SourceData F4 — is the source file for Fig. 4. [file JCB_202303102_SourceDataF4.pdf]

# Source data of Figure 5

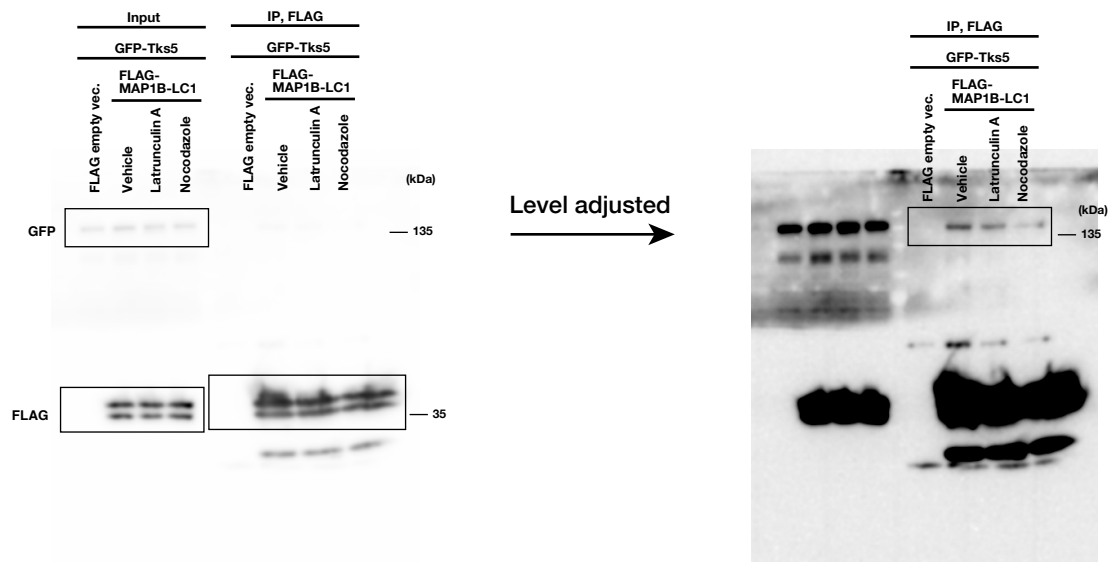

Supplement: SourceData F5 — is the source file for Fig. 5. [file JCB_202303102_SourceDataF5.pdf]

Source data of Figure 6

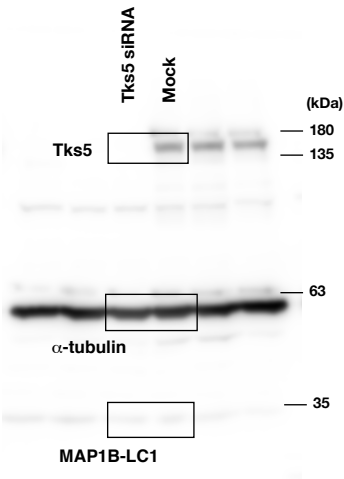

Supplement: SourceData F6 — is the source file for Fig. 6. [file JCB_202303102_SourceDataF6.pdf]

Source data of Figure 7

A

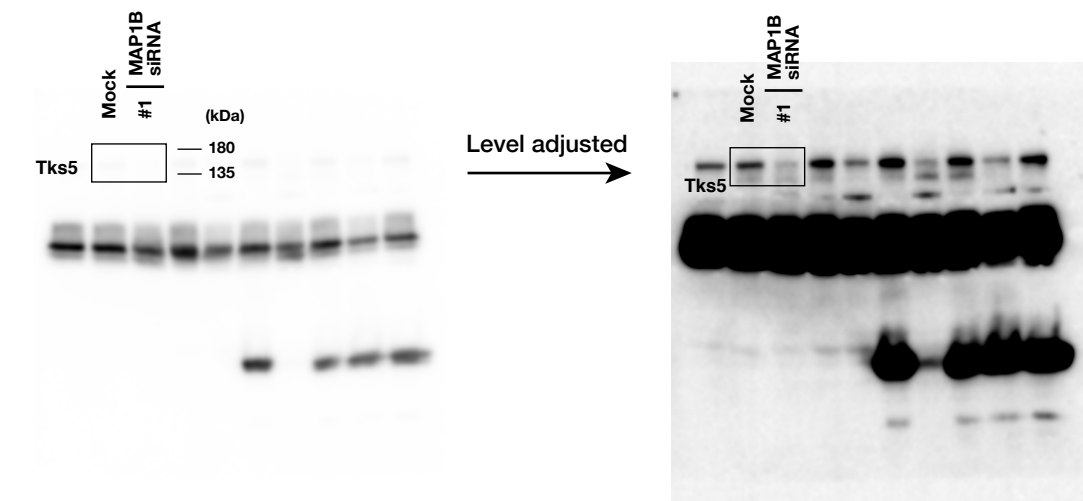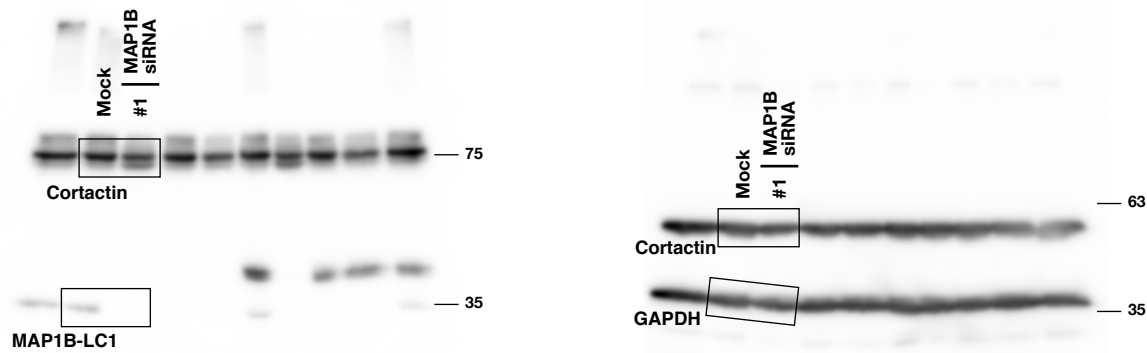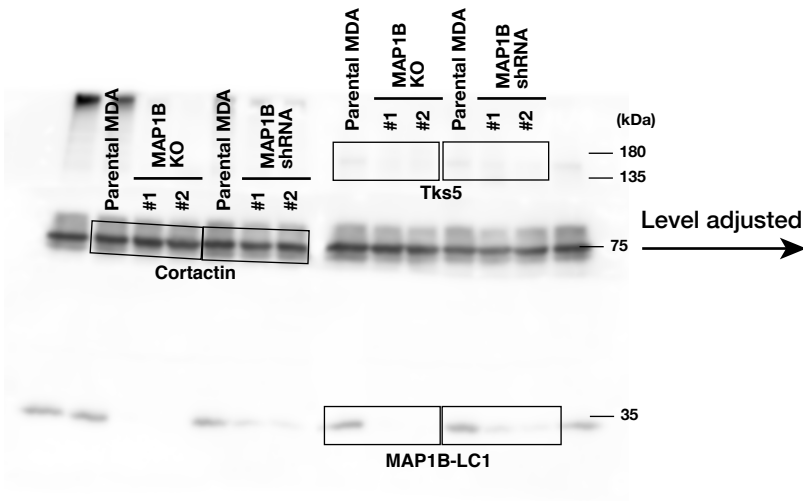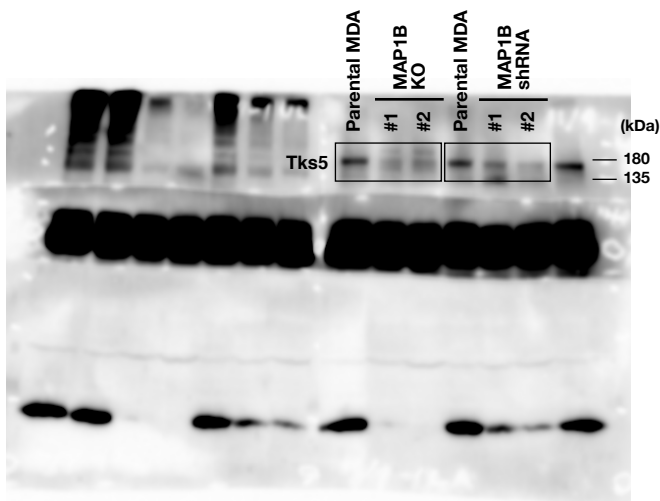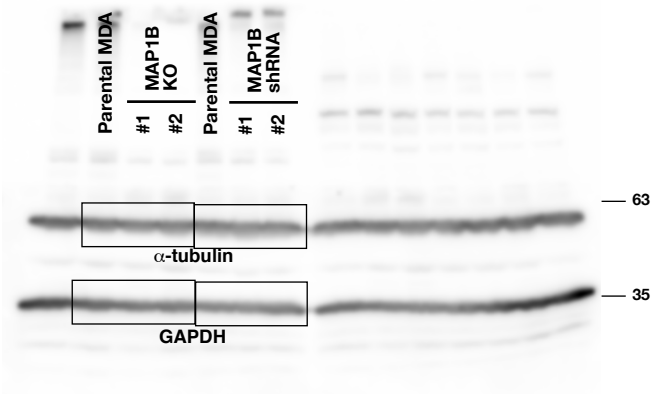

## Source data of Figure 7

**B**

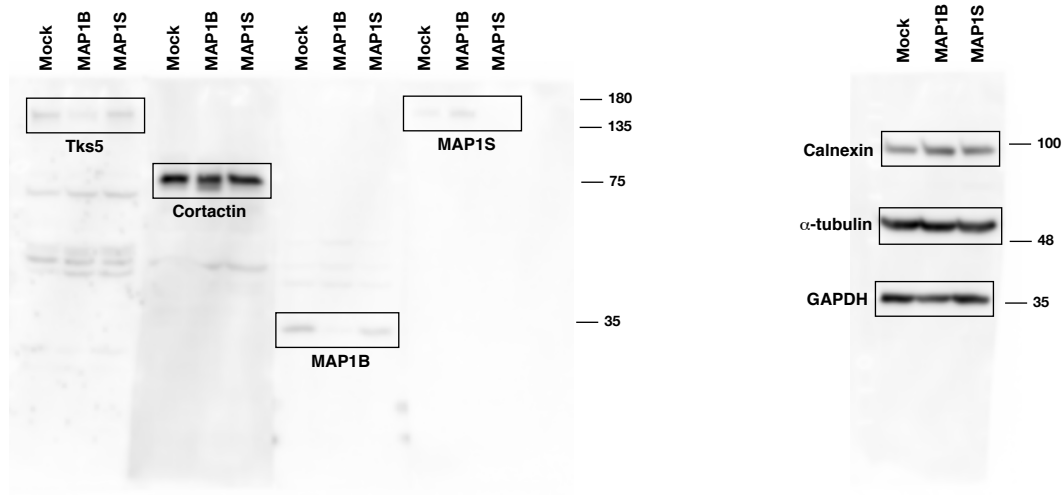

**D**

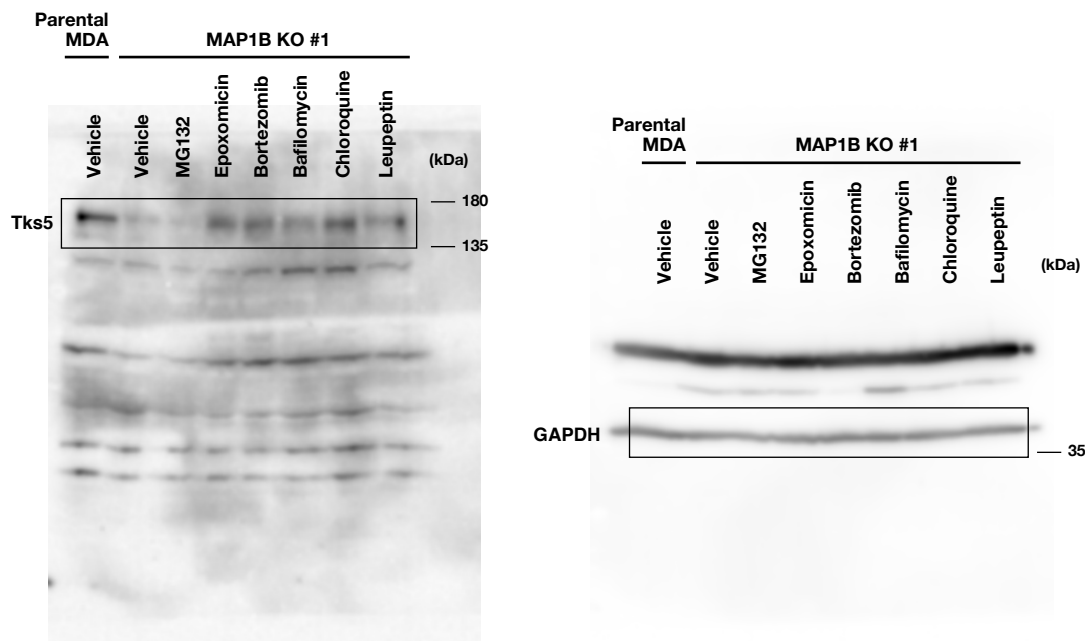

Source data of Figure 7

H

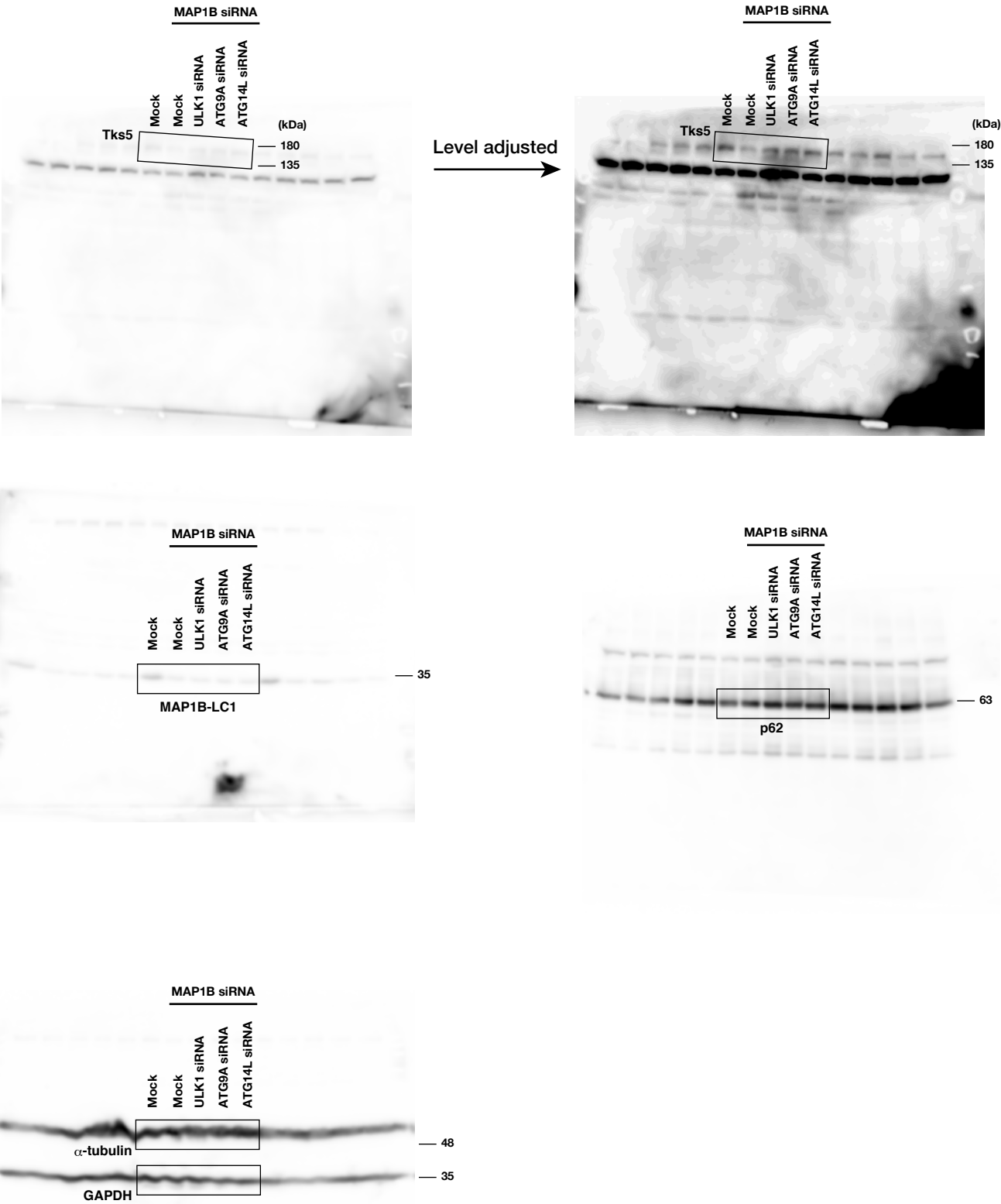

Supplement: SourceData F7 — is the source file for Fig. 7. [file JCB_202303102_SourceDataF7.pdf]
