# Supplementary material for: A MAP1B–cortactin–Tks5 axis regulates TNBC invasion and tumorigenesis
Source: J Cell Biol. 2024 Feb 14;223(3):e202303102. doi: 10.1083/jcb.202303102 (PMC10866687; doi:10.1083/jcb.202303102)

Source data of Supplementary Figure S4

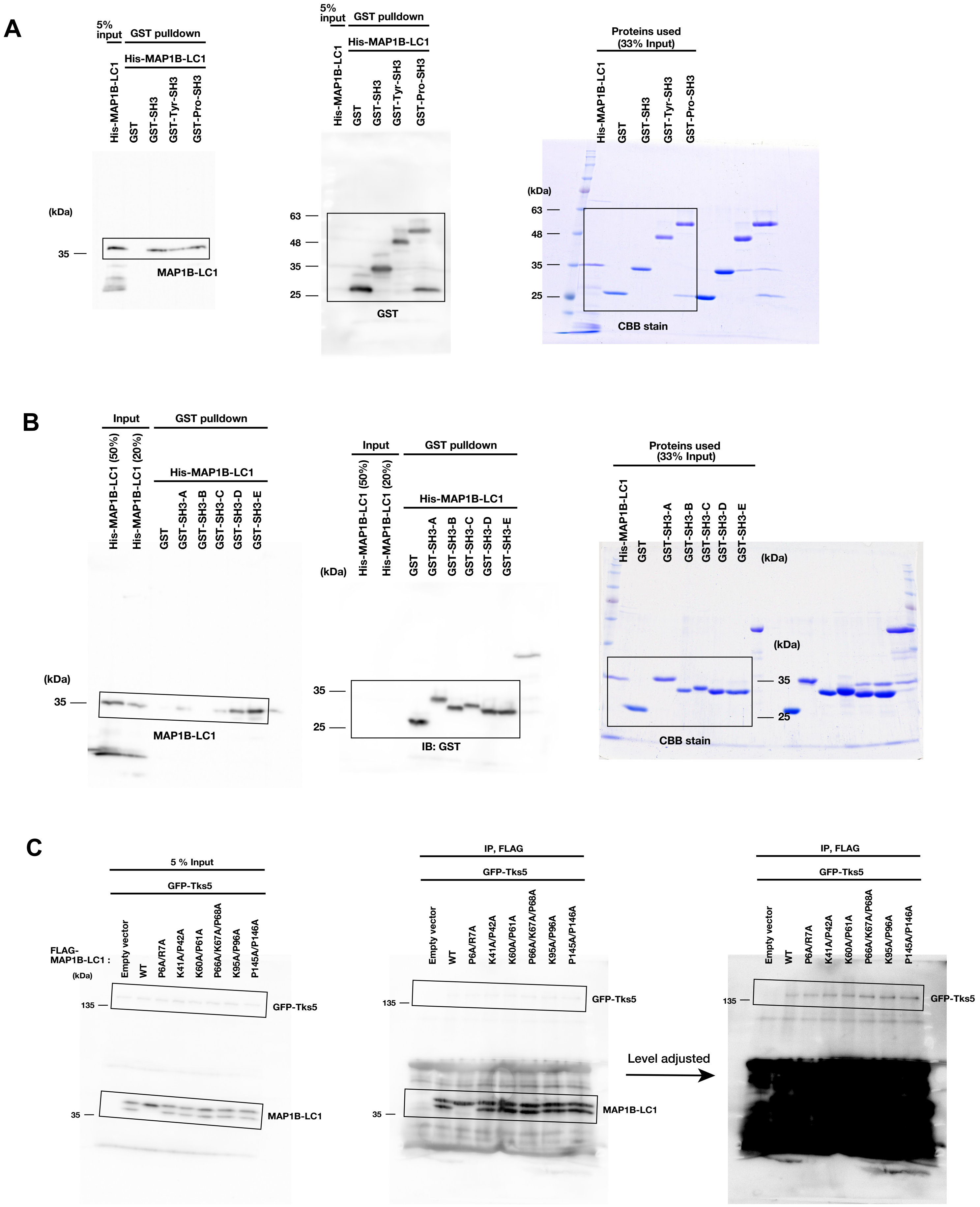

Source data of Supplementary Figure S4

D

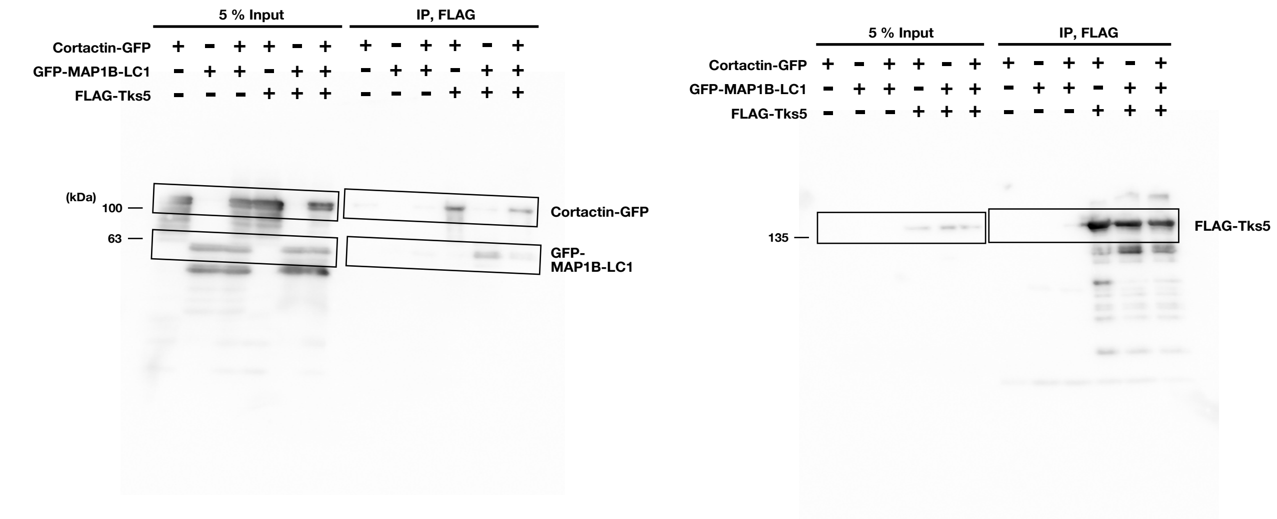

E

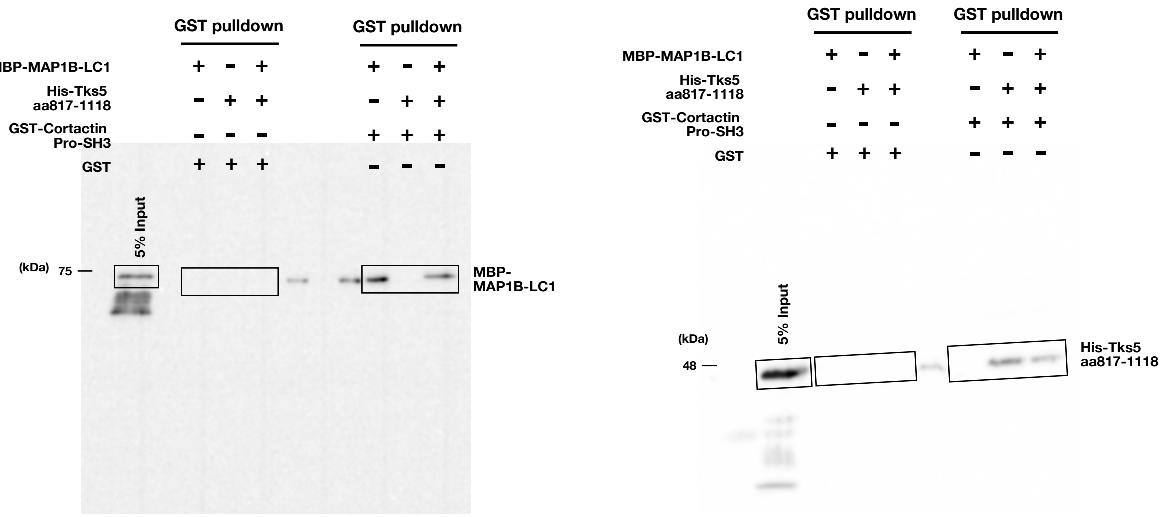

Source data of Supplementary Figure S4

H

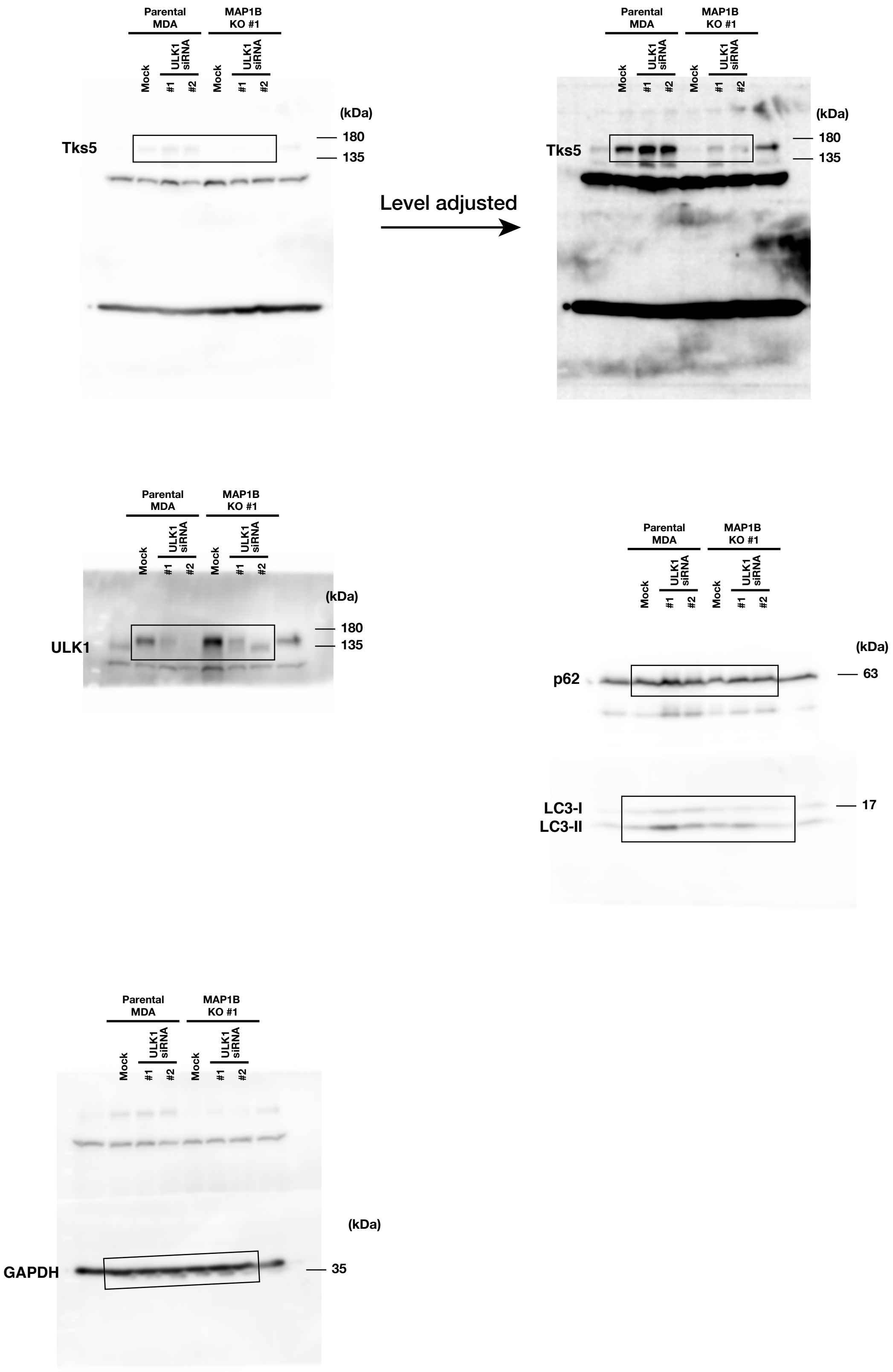

Supplement: SourceData FS4 — is the source file for Fig. S4. [file JCB_202303102_SourceDataFS4.pdf]
